# Trigeminal Contributions to the Dorsal Cochlear Nucleus in Mouse

**DOI:** 10.3389/fnins.2021.715954

**Published:** 2021-07-28

**Authors:** Timothy S. Balmer, Laurence O. Trussell

**Affiliations:** Vollum Institute and Oregon Hearing Research Center, Oregon Health & Science University, Portland, OR, United States

**Keywords:** dorsal cochlear nucleus, trigeminal, granule cell, mouse, auditory

## Abstract

The dorsal cochlear nucleus (DCN) is the first site of multisensory integration in the auditory pathway of mammals. The DCN circuit integrates non-auditory information, such as head and ear position, with auditory signals, and this convergence may contribute to the ability to localize sound sources or to suppress perceptions of self-generated sounds. Several extrinsic sources of these non-auditory signals have been described in various species, and among these are first- and second-order trigeminal axonal projections. Trigeminal sensory signals from the face and ears could provide the non-auditory information that the DCN requires for its role in sound source localization and cancelation of self-generated sounds, for example, head and ear position or mouth movements that could predict the production of chewing or licking sounds. There is evidence for these axonal projections in guinea pigs and rats, although the size of the pathway is smaller than might be expected for a function essential for a prey animals’ survival. However, evidence for these projections in mice, an increasingly important species in auditory neuroscience, is lacking, raising questions about the universality of such proposed functions. We therefore investigated the presence of trigeminal projections to the DCN in mice, using viral and transgenic approaches. We found that the spinal trigeminal nucleus indeed projects to DCN, targeting granule cells and unipolar brush cells. However, direct axonal projections from the trigeminal ganglion itself were undetectable. Thus, secondary brainstem sources carry non-auditory signals to the DCN in mice that could provide a processed trigeminal signal to the DCN, but primary trigeminal afferents are not integrated directly by DCN.

## Introduction

Accurate sound localization is essential for an animal’s survival and much of the auditory brainstem is specialized for this function. The dorsal cochlear nucleus (DCN), one of the first central targets of cochlear input, is thought to compute a sound source by integrating auditory spectral cues with multisensory (non-auditory) information regarding the position of the head and ears from motor, somatosensory, proprioceptive, and higher level auditory processing regions (Ryugo et al., 2003). However, the sources of multisensory information are not well understood, especially in mice, a species which has become an important model in auditory neuroscience.

The trigeminal pathway is likely to contribute to sound source localization. In principle, somatosensory signals from the head and face that could inform the auditory system of the current position of the jaw and ears – especially relevant to sounds source localization in animals with mobile pinna – are transmitted into the brainstem via the trigeminal pathway. This pathway carries cutaneous mechanosensory information from the face and head via first-order neurons of the trigeminal ganglion to the trigeminal brainstem nuclei. From the brainstem, the second-order neurons extend their axons to the contralateral thalamus along the trigeminal lemniscus. Disruption of this pathway may underlie some forms of tinnitus, the phantom percept of high-frequency sound commonly referred to as “ringing” of the ears. Somatic tinnitus has been linked to pathological enhancement of trigeminal input to DCN. Injury to the multisensory pathways that are thought to send signals to DCN can lead to tinnitus (Folmer and Griest, 2003). Intriguingly, in 80% of tinnitus patients, head, jaw, and neck movements can modulate the perception of tinnitus (Levine et al., 2003). These movements cause altered activity in DCN (Lanting et al., 2010), possibly by enhancing somatosensory input via the trigeminal pathway. Identifying the neurons involved in carrying these trigeminal signals to the auditory system will be crucial to understanding the neural mechanisms of somatic tinnitus.

The pathway that trigeminal signals take to arrive at the DCN is unclear. Specifically, whether the trigeminal ganglion projects axons directly to neurons in the cochlear nucleus or whether a polysynaptic pathway via the brainstem is the sole route is not understood and may depend on the species in question. This polysynaptic trigeminal ganglion – trigeminal nucleus – cochlear nucleus pathway has been demonstrated conclusively in cats (Itoh et al., 1987), rats (Haenggeli et al., 2005), and guinea pigs (Zhou and Shore, 2004; Zhou et al., 2007; Zeng et al., 2011). A remarkable study in mice recently revealed that these trigeminal nucleus to DCN projections were necessary to reduce the response of DCN neurons to self-generated sounds (Singla et al., 2017). This work indicated that second-order trigeminal inputs are processed by the cochlear nucleus in mice, but whether first-order trigeminal ganglion inputs also project to DCN is unknown.

The direct pathway from the trigeminal ganglion to the cochlear nucleus is less well supported. One anatomical tracing study in guinea pigs reported that the trigeminal ganglion sends axons to the small cell cap at the dorsal edge of the ventral cochlear nucleus (VCN) (Shore et al., 2000). In rats, however, no projection to the cochlear nucleus was reported in a similar anatomical tracing study (Marfurt and Rajchert, 1991). Electrophysiological studies in guinea pigs report latencies between trigeminal stimulation and effects on DCN or VCN neurons of >5 ms (Shore et al., 2003; Shore, 2005), which may be monosynaptic or polysynaptic. To our knowledge, a direct projection from the trigeminal ganglion to the cochlear nucleus has not been reported in other species. Here we examined the direct and indirect trigeminal pathways in mice using newly developed viral tracing technologies and report that direct projections from the trigeminal ganglion to DCN were below the level of detection, whereas the indirect projection via brainstem trigeminal nuclei was present and therefore may underly the integration of head, jaw, face, and ear signals in the auditory system, and may be related to somatic tinnitus.

## Results

### Trigeminal Nucleus Projections to DCN and Other Targets

**FIGURE 1:**
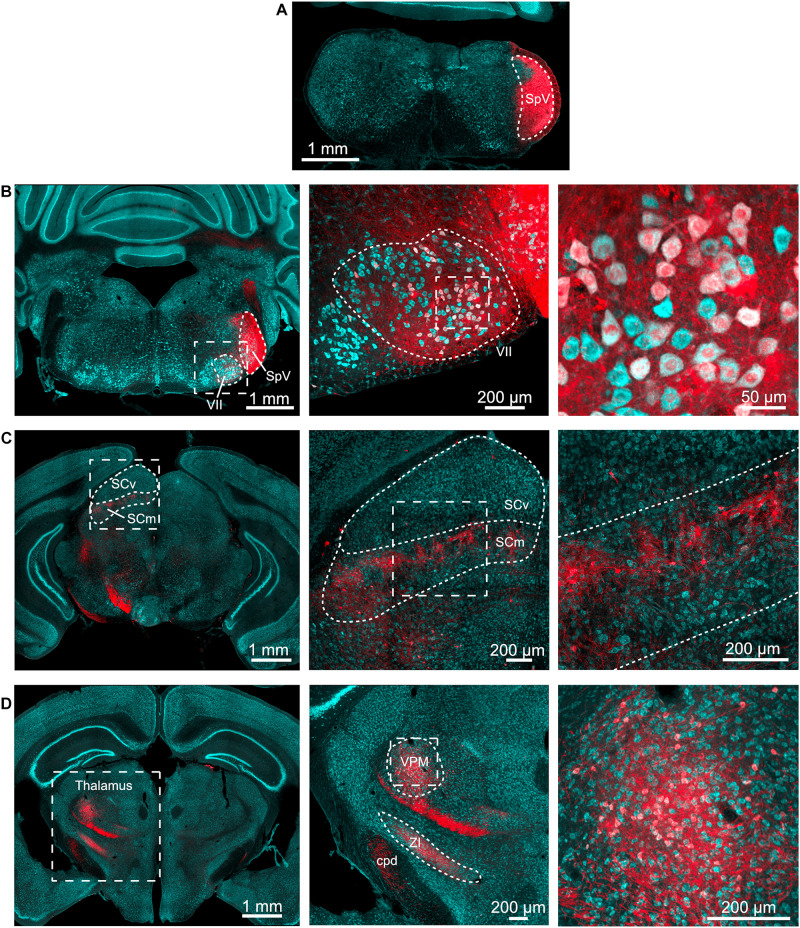
Spinal trigeminal nucleus injection labels expected targets. **(A)** AAV1-Syn-Cre (200 nl) was injected into the SpV of tdTomato-reporter (Ai9) mice. This is the injection site. Cyan – Nissl and red – tdTomato. **(B)** Neurons in the ipsilateral facial motor nucleus (VII) were labeled. Outline of SpV shown includes the tracts to its lateral side. Middle, right, magnified view of facial motor nucleus. **(C)** Neurons in the motor-related areas of the contralateral superior colliculus were labeled. SCv, superior colliculus, visual layers; SCm, superior colliculus, multisensory layers. Middle, right, magnified views of the boxed regions in panel to left. **(D)** Neurons in the thalamus were labeled. Middle, right, magnified views of the boxed regions in panel to left. ZI, zona incerta; VPM, ventral posteromedial nucleus of the thalamus; cpd, cerebellar peduncle. All images are from the same animal, although similar results were obtained from four animals with injection volumes varying from 100 to 500 nl.

To determine the projection pattern of trigeminal brainstem regions, we utilized an AAV1-Syn-Cre virus that is transferred from neurons at the injection site to their postsynaptic targets. After this monosynaptic anterograde transfer, the virus expresses Cre recombinase, which leads to the expression of a fluorescent protein in the Ai9 tdTomato reporter mouse (Zingg et al., 2017, 2020). AAV1-Syn-Cre was injected into the spinal trigeminal nucleus (SpV) of Ai9 mice (Figure 1A), and the axonal projections of the infected neurons were traced to their postsynaptic partners, as shown by clearly labeled tdTomato positive fibers and cell bodies in the facial motor nucleus, superior colliculus, and thalamus (Figures 1B–D). This pattern of expression confirms that SpV both projects to and makes synaptic contacts in these regions. While this result is expected, it verifies the efficacy of the transsynaptic labeling approach. While it is in principle possible for this approach to also label these circuit components in the reverse direction (with SpV as the postsynaptic target), such connectivity is not apparent in the Allen Brain Atlas database^1^.

**FIGURE 2:**
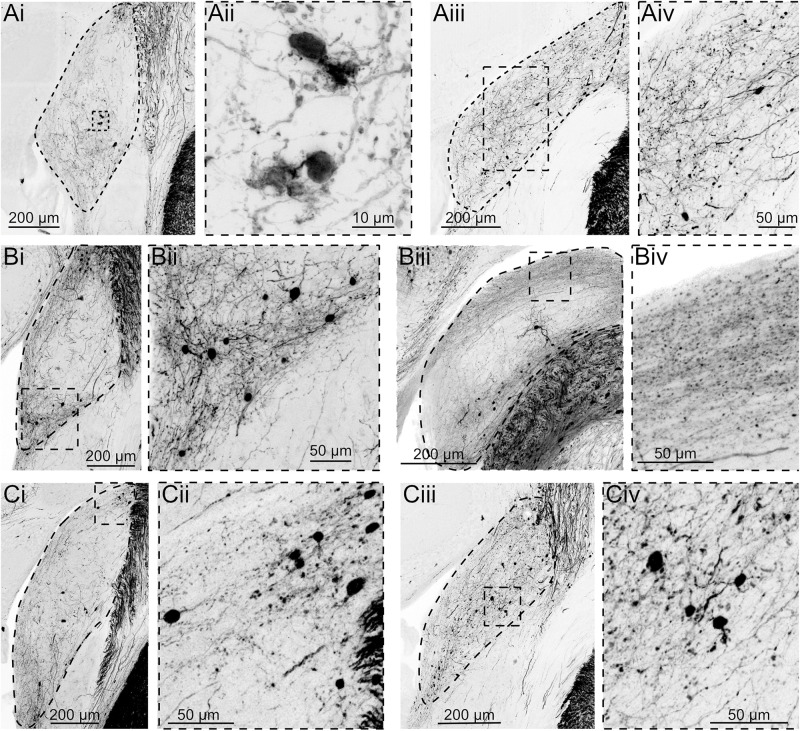
AAV1-Syn-Cre injection into SpV reveals projections to granule domain cells in DCN. Three different experiments, each with two example coronal sections containing DCN. tdTomato labeling is shown in black. DCN is outlined in the lower magnification images. **(Ai)** Although few neurons are transsynaptically labeled, UBCs can be identified by their distinct morphology. **(Aii)** Magnification of the boxed region in **Ai**. **(Aiii)** A caudal section containing DCN from the same animal. **(Aiv)** Magnification of the boxed region in **Aiii**. **(Bi)** Another experiment showing transsynaptically labeled small cells in the region between DCN and VCN. **(Bii)** Magnification of the boxed region in **Bi**. **(Biii)** A more caudal section of DCN demonstrates labeled parallel fiber axons, confirming that granule cells were labeled transsynaptically. **(Biv)** Magnification of the boxed region in **Biii**. **(Ci–iv)** A third experiment further demonstrating that a small but consistent population of neurons receives synaptic input from SpV projections.

In the same experiments, fibers and cell bodies were also observed in the DCN and adjacent granule cell domain of the cochlear nucleus, although the density of cells was markedly less than was observed in other SpV targets (Figure 2). The labeled postsynaptic cells included unipolar brush cells (UBCs), characterized by their brush dendrite (Figure 2Aii) and granule cells, identified by their small size and short, spindly dendrites (Figures 2Bii,Cii,Civ). Labeling in fibers of the DCN molecular layer (Figures 2Biii,iv), where parallel fibers accumulate, further confirms that granule cells are a target of SpV fibers. Thus, the SpV was confirmed to provide input to the DCN and granule cell domain, consistent with previous studies in mice (Singla et al., 2017).

### First-Order Trigeminal Nerve Projections to DCN

**FIGURE 3:**
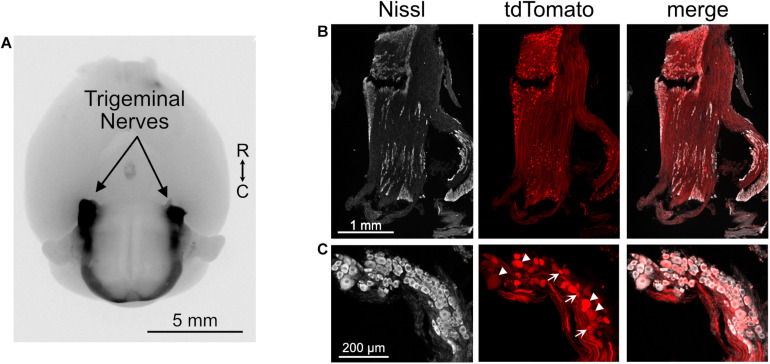
Trigeminal ganglion labeled with AAV.PHP-s virus. **(A)** Image of ventral aspect of the brain of a mouse that received a retro-orbital injection of AAV.PHP-s.CAG.tdTomato. The trigeminal nerves are brightly labeled, as are axons in the spinal cord that likely originate from dorsal root ganglia. tdTomato is shown in black. R, rostral; C, caudal. **(B)** Section of trigeminal ganglion demonstrating that many somata were labeled by this approach. **(C)** Neurons with a diverse range of soma sizes were labeled. Arrowheads – large somata and arrows – small somata.

To test the hypothesis that the trigeminal ganglion projects directly to DCN and granule cell domains, we utilized an engineered adeno-associated virus (AAV.PHP-s.CAG.tdTomato) that, when injected intravenously, infects and fluorescently labels the peripheral nervous system (Chan et al., 2017). This approach was favored over attempting to inject a virus into the trigeminal ganglion because it could more homogenously and intensely label all three branches of the trigeminal nerve. Somata of the trigeminal ganglion were well-labeled and their axons that make up the trigeminal nerves were clearly seen entering the brain (Figure 3A). Although not all of the trigeminal ganglion neurons were labeled, the variety of soma sizes that were labeled indicates that a diverse sample of cell types was infected by this virus (Figures 3B,C). We note that while the trigeminal ganglion was strongly labeled by this virus, auditory and vestibular ganglia were not labeled, either due to a lack of accessibility to the inner ear vasculature or due to specificity of the viral serotype.

**FIGURE 4:**
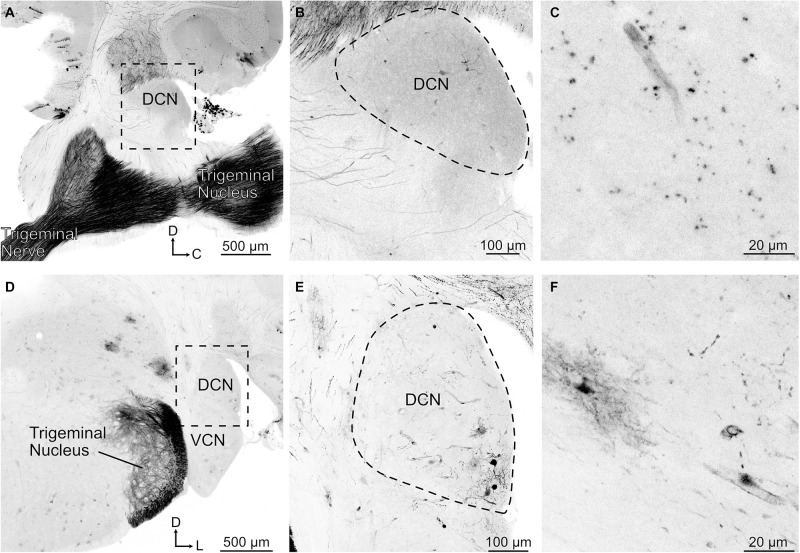
Trigeminal nerve makes few projections to DCN. **(A)** Sagittal section containing trigeminal nerve, trigeminal nucleus, and DCN. Note that there are few if any labeled axonal fibers that project to DCN despite strong labeling of nerve. tdTomato labeling is shown in black. D, dorsal; C, caudal. **(B)** Magnified view of the boxed region in **A**. **(C)** Higher magnification of a sagittal DCN section showing labeled blood vessel. **(D)** Coronal section showing trigeminal nerve fibers labeled in the trigeminal nucleus but not in the DCN. VCN, ventral cochlear nucleus; D, dorsal; L, lateral. **(E)** Magnified view of the boxed region in **D** showing that the labeled neurons are clustered around a blood vessel. **(F)** Higher magnification of a coronal DCN section showing a labeled blood vessel, glial cell, and few neuronal processes.

In the brainstem, SpV had a confluence of tdTomato positive fibers throughout the brainstem, extending well into the region of the cerebellar nuclei and cerebellar cortex (Figure 4A). The cerebellar labeling confirmed the presence of direct trigeminal projection to the cerebellum in mice (Marfurt and Rajchert, 1991), and shows that the expression of tdTomato in trigeminal fibers reaches far into the brain. However, in marked contrast to this dense labeling, trigeminal ganglion fibers in the cochlear nucleus were undetectable (Figures 4B,D). We conclude that the trigeminal ganglion does not directly target the cochlear nucleus of mice, including VCN, DCN, or granule cell regions. Occasional labeled cell bodies, probably cartwheel cells, were observed in the DCN near the molecular layer (Figure 4). However, this labeling is consistent with the sparse glia and neurons labeled throughout the brain primarily near the surface of the brain or near blood vessels, presumably due to the intravenous delivery of the virus (cerebellar Purkinje cells in Figure 4A). This AAV.PHP-s virus has not been reported to jump across synapses and we do not suspect that these labeled cells are postsynaptic to primary afferents. The fact that the sparse labeling of cells is not due to transsynaptic spread of virus is supported by the absence of somatic labeling within the regions of the trigeminal nuclei despite the presence of dense afferent fibers (Figure 4).

## Discussion

Mouse models for hearing disorders are increasingly common, due to the ready availability of genetic mutations that affect hearing, or enhance the accessibility of cellular elements for anatomical or physiological study (Ohlemiller, 2019). Recent studies of tinnitus have focused on alterations in the function of principal cells of the DCN that might be related to plasticity in the inputs from non-auditory sources associated sensation in the head and neck (Levine et al., 2003; Kaltenbach, 2006). The role of trigeminal input to granule cell domains in and around the cochlear nucleus has been proposed to be part of this modulatory pathway affected during tinnitus (Shore et al., 2007). In other species, stimulation of trigeminal inputs leads to spike modulation in DCN and VCN (Shore et al., 2003; Shore, 2005), but direct evidence for this in mice is lacking. In the present work, we sought to provide further support for the use of the mouse model by confirming anatomically trigeminal projections to the cochlear nucleus. Unlike previous anatomical studies, we used a viral approach to take advantage of the enhanced infection afforded by AAV reagents in the hope that a robust labeling would be obtained.

Indeed, injection into trigeminal nuclei of tdTomato reporter mice of an anterogradely transported Cre-expressing virus capable of transsynaptic labeling led to robust expression of labeled fibers and cells in numerous known trigeminal targets, including trigeminal nuclei, thalamus, superior colliculus, facial nucleus, and cerebellum. Fibers and cell bodies, including presumptive granule cells and unipolar brush cells, were also found in the granule cell lamina and cell body region of the DCN, although at an apparently lower density than in other trigeminal targets. Such labeling is consistent with a previous study in mice showing trigeminal nuclear input to DCN and its apparent necessity for modulation of the DCN’s response to self-generated sounds associated with licking (Singla et al., 2017).

In contrast to this result obtained by virus injection into a second-order nucleus, we were unable to confirm that primary trigeminal afferents project to the cochlear nucleus. Here, we used a systemically injected virus previously shown to be taken up in peripheral ganglia (Chan et al., 2017). Several observations confirmed that labeling with this approach was sufficient to label nearly all ganglionic inputs. Expression of the tdTomato reporter was obvious throughout the ganglion, the seventh cranial nerve, and its projections into the brain. Indeed, dense fibers were seen in trigeminal nuclei, as well as fibers extending into the cerebellum. The latter projection has been described previously and was considered “scant” as compared to secondary trigeminal projections (Marfurt and Rajchert, 1991), but was quite obvious in our micrographs.

However, no primary trigeminal afferent fibers were apparent in cochlear nucleus, in contrast to a previous report in guinea pig (Shore et al., 2000). We consider three reasons for these conflicting results. First, it may be that the difference in species is a factor. The hearing range of guinea pigs is somewhat lower than that of mice (Warfield, 1973; Fay, 1988), and perhaps the functional significance of convergence to the DCN from non-auditory sources varies as well. Second, it may be that the primary trigeminal input is sparse even in guinea pig, consistent with the micrographs shown in Shore et al. (2000). While trigeminal stimulation drives activity in guinea pig DCN, this response could largely reflect a disynaptic circuit. A third possibility is that the viral method we used here was somehow biased against infection of those ganglion somata that target the cochlear nucleus. However, regardless of the reasons, our results provide confirmation of trigeminal nuclear innervation of the cochlear nucleus and further support the use of the mouse as a model for somatosensory modulation of auditory function. Moreover, given that some targets of primary trigeminal afferents have also been shown to project to cochlear nucleus [dorsal horn of C1 and C2, ventral horn of cervical spinal cord, cuneate, and vestibular nuclei (Marfurt and Rajchert, 1991)], the trigeminal influence of the function of the cochlear nucleus may extend beyond the trigeminal nuclei themselves.

## Materials and Methods

### Animals

Ai9(RCL-tdT) (Madisen et al., 2012) or C57BL/6J mice of both sexes were bred in-house and all procedures were approved by the Oregon Health and Science University’s Institutional Animal Care and Use Committee.

### Intracranial Viral Injections

Viral injections were made into the SpV of four adult mice (>3 months old) using a stereotax (Kopf), single-axis manipulator (Narishige), and pipette vice (Ronal) under isoflurane anesthesia. Glass capillaries (Drummond Scientific) were pulled on a pipette puller (Sutter P-97) and beveled at 45° angle with a 20–30 μm inside diameter using a diamond lapping disk (3M). An incision was made in the scalp along the midline, and a small hole was drilled into the skull. The pipette was lowered into the brain at 10 μm/s. Five-minute periods were allowed before and after injection. AAV1-Syn-Cre (3.15e13 GC/ml) was purchased from the University of Pennsylvania’s viral vector core. In total, 100–500 nl of undiluted virus was injected using stereotaxic coordinates (7.8 mm caudal, 2.2 mm lateral, 3.5 mm ventral, relative to bregma).

### Intra-Orbital Viral Injections

Two P18–30 mice were anesthetized with isoflurane and 5–30 μl of the AAV.PHP-s.CAG.tdTomato virus was injected into the retro-orbital sinus using a 0.5-ml syringe with a 28 ga needle. The AAV.PHP-s.CAG.tdTomato (1.7e13 GC/ml) was purchased form Addgene.

### Immunohistochemistry and Imaging

Three weeks after viral injection, mice were overdosed with isoflurane and perfused through the heart with 0.01 M phosphate buffered saline, 7.4 pH (PBS) followed by 4% paraformaldehyde in PBS. Brains and trigeminal ganglia were extracted from the skull and incubated in the same solution overnight at 4°C. Fifty-micrometer-thick sections were made on a vibratome and saved as floating sections in PBS. Sections were rinsed 3 × 10 min in PBS, blocked, and permeabilized in 5% normal donkey serum (NDS), 2% fish gelatin, and 0.2% Triton X-100 in PBS for >2 h at room temperature. Sections were incubated in primary antibodies to amplify the tdTomato labeling using 1:400 rabbit anti-DsRed (632496, Clontech) in 5% NDS for 2–3 days at 4°C on an orbital shaker. Sections were rinsed 3 × 10 min in PBS, followed by 1:500 donkey anti-rabbit Cy3 (711-165-153, Jackson ImmunoResearch) in 5% NDS for 2–3 days at 4°C on an orbital shaker. The sections were mounted on microscope slides and, in some cases, a fluorescent nissl stain (1:50 NeuroTrace 435/455, Invitrogen) was applied for 0.5–2 h. The slides were coverslipped with Fluoromount-G (Southern Biotech). Images were acquired on a confocal microscope (Zeiss 780 or 880) or on a Zeiss Elyra PS.1 with AiryScan system that reconstructs super-resolution images from a series of images acquired under spatially structured illumination (Gustafsson, 2000). In some cases, tdTomato labeling was converted to grayscale and then inverted in order to enhance the contrast of the fluorescent labeling. All images are maximum intensity projections.

## Data Availability Statement

The raw data supporting the conclusions of this article will be made available by the authors, without undue reservation.

## Ethics Statement

The animal study was reviewed and approved by the Institutional Animal Care and Use Committee of OHSU.

## Author Contributions

LT and TB conceived the experiments and wrote the manuscript. TB conducted the experiments and analyzed the data. Both authors contributed to the article and approved the submitted version.

## Conflict of Interest

The authors declare that the research was conducted in the absence of any commercial or financial relationships that could be construed as a potential conflict of interest.

## Publisher’s Note

All claims expressed in this article are solely those of the authors and do not necessarily represent those of their affiliated organizations, or those of the publisher, the editors and the reviewers. Any product that may be evaluated in this article, or claim that may be made by its manufacturer, is not guaranteed or endorsed by the publisher.

## Footnotes

1
https://connectivity.brain-map.org/
